# Acclimation and Institutionalization of the Mouse Microbiota Following Transportation

**DOI:** 10.3389/fmicb.2018.01085

**Published:** 2018-05-28

**Authors:** Dan R. Montonye, Aaron C. Ericsson, Susheel B. Busi, Cathleen Lutz, Keegan Wardwell, Craig L. Franklin

**Affiliations:** ^1^Comparative Medicine Program, Department of Veterinary Pathobiology, University of Missouri, Columbia, MO, United States; ^2^University of Missouri Metagenomics Center, University of Missouri, Columbia, MO, United States; ^3^University of Missouri Mutant Mouse Resource and Research Center, University of Missouri, Columbia, MO, United States; ^4^The Jackson Laboratory, Bar Harbor, ME, United States

**Keywords:** mouse models, gut microbiota (GM), transportation, acclimation, 16S rRNA gene sequencing

## Abstract

Using animal models, the gut microbiota has been shown to play a critical role in the health and disease of many organ systems. Unfortunately, animal model studies often lack reproducibility when performed at different institutions. Previous studies in our laboratory have shown that the gut microbiota of mice can vary with a number of husbandry factors leading us to speculate that differing environments may alter gut microbiota, which in turn may influence animal model phenotypes. As an extension of these studies, we hypothesized that the shipping of mice from a mouse producer to an institution will result in changes in the type, relative abundance, and functional composition of the gut microbiota. Furthermore, we hypothesized that mice will develop a microbiota unique to the institution and facility in which they are housed. To test these hypotheses, mice of two strains (C57BL/6J and BALB/cJ), two age groups (4 week and 8 week old), and originating from two types of housing (research animal facility under conventional housing and production facilities under maximum barrier housing) were obtained from The Jackson Laboratory. Fecal samples were collected the day prior to shipping, immediately upon arrival, and then on days 2, 5, 7, and weeks 2, 4, and 9 post-arrival. Following the first post-arrival fecal collection, mice were separated into 2 groups and housed at different facilities at our institution while keeping their caging, diet, and husbandry practices the same. DNA was extracted from the collected fecal pellets and 16S rRNA amplicons were sequenced in order to characterize the type and relative abundance of gut bacteria. Principal component analysis (PCA) and permutational multivariate analysis of variance (PERMANOVA) demonstrated that both the shipping and the institution and facility in which mice were housed altered the gut microbiota. Phylogenetic investigation of communities by reconstruction of unobserved states (PICRUSt) predicted differences in functional composition in the gut microbiota of mice based on time of acclimation.

## Introduction

The transportation of laboratory mice from producers and animal facilities is a common occurrence in research. Most animal distributors take great care in ensuring that proper housing and environmental conditions are maintained throughout the shipping process, but even with strict guidelines to ensure animal welfare, previous studies have shown that the shipping process can induce numerous physiologic changes that are indicative of stress in rodents (Landi et al., 1982; Aguila et al., 1988; van Ruiven et al., 1998; Swallow et al., 2005; Capdevila et al., 2007; Laroche et al., 2009; Shim et al., 2009; Arts et al., 2012; Lee et al., 2012). From an animal welfare standpoint, it is important to recognize that these stress-like responses in animals do not necessarily indicate a pathologic change. Instead, they can simply indicate a normal response from the animal to maintain physiologic homeostasis. From a research standpoint however, it is important to recognize that these physiologic and behavioral responses to stressors can potentially impact study results if not taken into consideration.

Previous studies have shown that many alterations that occur in response to transportation normalize within 48–72 h following arrival, although this can be highly variable (Ruiven et al., 1996; Conour et al., 2006; Obernier and Baldwin, 2006). Because of these findings, many institutions have adopted mandatory acclimation periods upon receiving transported mice that commonly range from 48 to 72 h. However, as addressed in the Guide for Humane Transportation of Research Animals (National Research Council, 2006), many factors such as age, strain, and length of shipment can extend the time required for normalization to weeks, and it is critical that investigators consider the effects of transportation in relation to their specific animal model.

One physiologic change that has yet to be thoroughly investigated in regards to acclimation following transportation is the gut microbiota (GM) of mice. Currently, there are limited data on how the GM of mice is altered by contemporary shipping methods, and how long it takes to normalize following transportation. Prior research has shown that moving mice from a specific pathogen-free (SPF) status facility to a conventional facility within the same institution results in a transient shift in the GM of mice that required 5 days to return to baseline (Ma et al., 2012). However, there is limited data demonstrating the changes after a more extended transport, which frequently occurs when obtaining mice from a commercial vendor, accredited repository, or another research institution. As it is becoming increasingly evident that differences in GM can have significant impacts on the disease and behavior phenotypes of mouse models (Moloney et al., 2014; Hart et al., 2017), this is particularly concerning. Knowing how the GM changes in mice following extended transportation will be critical to improving accuracy and reproducibility in studies using mouse models.

As the shipping process may involve maternal separation, acoustic stress, crowding, heat stress, and changes in light cycles, all of which have been shown to modulate the GM (Tannock and Savage, 1974; Suzuki et al., 1983; Bailey and Coe, 1999; O'Mahony et al., 2009; Bailey et al., 2010, 2011; Moloney et al., 2014; Rea et al., 2016), the aim of this study was to determine the extent and duration of alterations in the GM of mice associated with shipment from a mouse repository (The Jackson Laboratory). The Jackson Laboratory is an NIH approved vendor and as such, maintains the highest of standards in shipping animals. All mice are shipped in protective filtered containers with dedicated, climate controlled transportation, with regulations on housing density to minimize stress. Because previous work in our laboratory has shown that mice of similar genetic background but from different producers harbor a GM unique to each producer (Ericsson et al., 2015), we aimed to show that individual research institutions could also drive the GM of mice to be distinct following shipment and acclimation to a new facility. Additionally, we hypothesized that mice kept in separate facilities within an institution may experience changes of their GM based on the facility in which they are housed. For these reasons, mice that were shipped for this study were divided between two separate facilities following arrival in order to assess the amount of intra-institutional facility-dependent influence on the GM. In an effort to minimize GM changes due to factors already recognized or suspected to influence the GM of research animals, mice that were received were housed under similar husbandry conditions keeping caging, bedding, food, water source, lighting, and care staff the same between facilities.

As previously mentioned, age and strain are two common factors that can alter normalization of physiologic parameters following shipping. Thus, we examined both C57BL/6 and BALB/c mice, two of the most commonly used strains in biomedical research, as well as adult and weanling C57BL/6J mice. Additionally, to determine whether housing conditions at the distributing institution prior to transportation influenced susceptibility to shipping-associated changes in the GM, studies were performed using both barrier and conventional-bred mice. Lastly, shipments occurred at different points in the year to identify if results were repeatable and not wholly dependent on one instance of transportation.

Given the recent advocacy by organizations like the NIH to improve reproducibility in biomedical research (Collins and Tabak, 2014), it is imperative that investigators understand the role that alterations in the GM may have on the phenotype of their animal models. Establishing control measures, such as time to acclimation following transportation, will help prevent unwanted and unidentified variables in the research that involves them. Understanding that mice housed at separate institutions may harbor distinctly different GMs, even if obtained from the same supplier, will also highlight some of the challenges associated with reproducibility of mouse models. While discrepancies in model phenotypes between institutions are often considered a source of frustration, it can also be an intriguing tool for scientific discovery. Indeed, if alterations in the GM are driving phenotypic variation, recognizing and then identifying the mechanism by which the GM drives this variation could lead to new treatment modalities targeting the GM.

## Materials and methods

### Study design and husbandry

**Figure 1:**
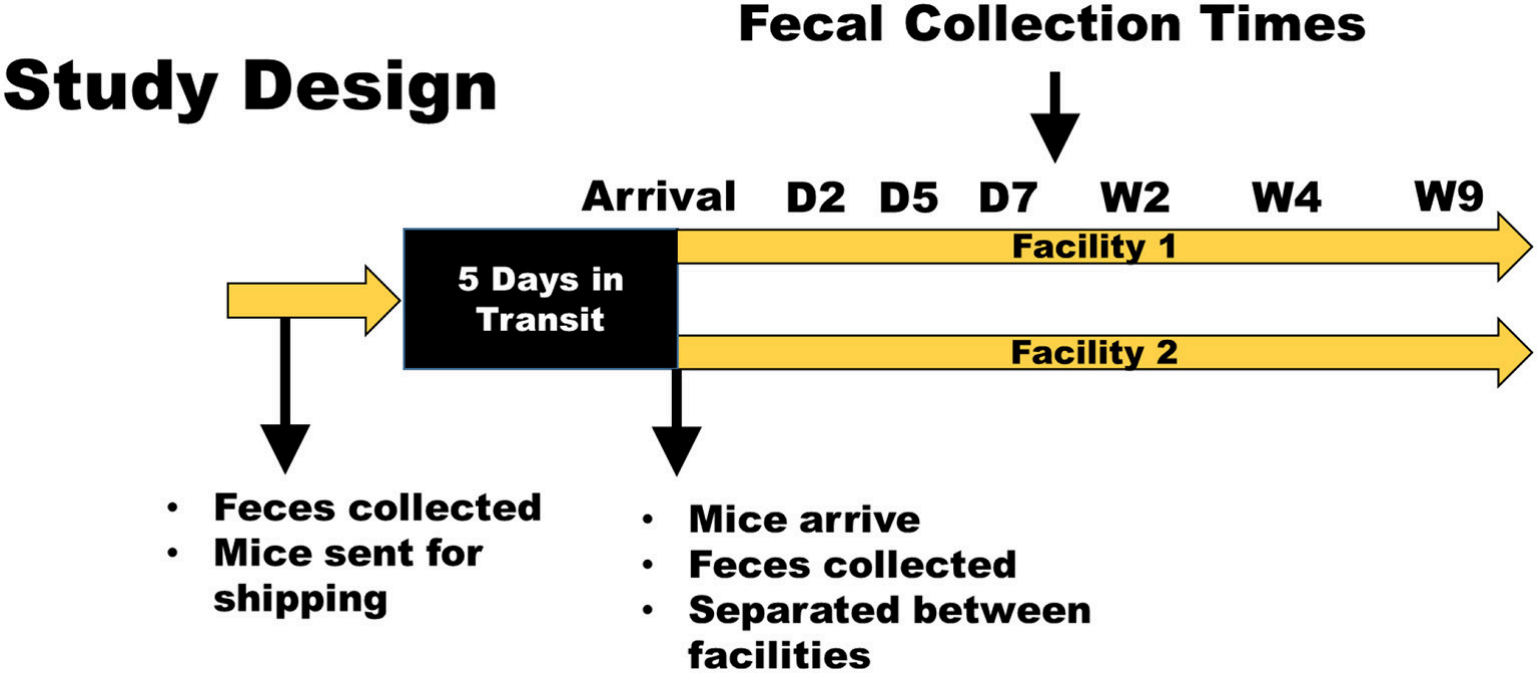
Study design and timeline. Mice were obtained from The Jackson Laboratory and delivered to two facilities at the University of Missouri. Fecal samples were obtained prior to shipping, at arrival, and at days 2, 5, and 7, and weeks 2, 4, and 9. Husbandry conditions (caging, bedding, food, water, carestaff, etc.) were kept the same between groups and between facilities. A second shipment was repeated in this same manner ~7 months following the first shipment to determine if seasonal differences also play a role in the GM response to transportation and acclimation.

An outline of study design and the timeline of events is shown in Figure 1. Five groups of eight mice were examined for this study. Groups of mice were co-housed and randomly assigned to their cage and facility. As this co-housing and rearrangement can often lead to fighting amongst males, which could confound and add to the stress-related changes from shipping, only females were used for this study. Mice raised in the conventional caging from the research animal facility at The Jackson Laboratory were housed together and all came from the same room, as did mice raised in the production level, maximum barrier rooms. Mice in both conventional and barrier statuses were housed on aspen chip bedding and fed LabDiet of 5K52/5K67 prior to shipping. The first shipment (February 2015) consisted of 8-week-old (adult), C57BL/6J (B6J) mice raised in a conventional (CON) room. Other groups examined in that shipment were adult BALB/cJ (CJ) CON mice, and adult B6J mice raised in a barrier (BAR) room at the Jackson Laboratory. Another shipment (September 2015), occurring ~7 months later, examined another group of adult B6J conventionally raised mice (CON2), as well as a group of 4-week-old (weanling) B6J CON mice. Fecal samples were collected from all mice at The Jackson Laboratory prior to transportation and shipped overnight on dry ice. Mice were placed in their transportation containers the same day and ear notches were used to match individual mice to fecal samples upon arrival. For both shipments, mice spent 5 days in transit. After feces was collected upon arrival, the mice were divided equally between two different facilities and co-housed in groups of four. Mice were housed at either the Discovery Ridge vivarium (facility 1) or the Animal Sciences Research Center vivarium (facility 2) at the University of Missouri, and all procedures were performed under the approval of the University of Missouri Institutional Animal Care and Use Committee and according to the guidelines of the Guide for the Care and Use of Laboratory Animals. Further fecal samples were obtained on post arrival days 2, 3, 5, 7, and weeks 2, 4, and 9. In both facilities, mice were housed conventionally in polycarbonate microisolater cages with compressed pelleted paper bedding (Thoren, Hazeleton, PA) and nestlets (Ancare, Bellmore, NY) on static racks with *ad libitum* access to irradiated chow (Labdiet 5008, LabDiet, St. Louis, MO) and acidified, autoclaved water, under a 14:10 light/dark cycle. Cages were changed, cleaned, and setup weekly by the same care staff for both facilities. The cages, chow, water, and bedding were obtained from the same source for both locations. The only notable differences in the housing conditions between locations were the physical rooms themselves (with minor variations in ambient light levels and temperature and humidity), and the presence of different background strains of mice also housed within the rooms.

### Sample collection

Individual mice were placed in empty autoclaved cages and monitored for defecation. Samples were collected using autoclaved toothpicks and placed in sterile 2 mL round-bottom tubes containing a 0.5 cm diameter stainless steel bead. All samples, including the arrival samples, were collected in the morning between the hours of 7 a.m. and 10 a.m. Once collected, samples were stored in −80°C freezers until DNA extraction took place.

### DNA extraction

All DNA extraction was performed as previously described (Ericsson et al., 2015). Lysis buffer (800 μL containing 4% sodium dodecyl sulfate, 500 mM NaCl, and 50 mM EDTA) was added to the 2 mL round-bottom tubes containing a stainless steel bead, and was then homogenized with a TissueLyser II (Qiagen, Venlo, Netherlands). Tubes were incubated at 70°C for periodic vortexing and then centrifuged at 5,000 × g for 5 min at room temperature. This was then transferred to a clean l.5 mL Eppendorf tube. 10 M ammonium acetate (200 μL) was added to the collected supernatant and then allowed to incubate on ice for 10 min. This was then centrifuged at 16,000 × g for 10 min at room temperature. After removal of the supernatant, an equivalent volume of isopropanol was added, mixed, and allowed to incubate on ice for 30 min. Nucleic acids were then pelleted at 16,000 × g for 15 min at 4°C. The pellets were then rinsed with 70% ethanol twice and resuspended in Tris-EDTA. They were then purified with DNeasy kits (Qiagen) according to manufacturer's instructions. A Quibit 2.0 fluorometer and Quibit dsDNA BR assay kits (Invitrogen) were used to measure DNA yields according to manufacturer's instructions.

### 16s rRNA library preparation and sequencing

Extracted DNA (100 ng) was processed at the University of Missouri DNA Core Facility as previously described (Ericsson et al., 2015). Briefly, an amplicon library of the V4 region of the 16S rRNA gene was generated using normalized DNA as a template. Using single-indexed (Walters et al., 2011) universal primers (U515F/806R) (Caporaso et al., 2011) flanked by Illumina adapter sequences with PCR parameters of 98°C^(3m)^ + [98°C^(15s)^ + 50°C^(30s)^ + 72°C^(30s)^] × 25 cycles + 72°C^(7m)^, amplicons were generated. These were then pooled for sequencing using Illumina MiSeq and V2 chemistry with 2 × 250 base pair pared-end reads.

### Informatics processing

Trimming, assembly, binning, and annotation of contiguous sequences was performed at the University of Missouri Informatics Research Core Facility. Quality control of DNA was performed by fragment analyzer on the pooled amplicon library to confirm correct amplicon size (~430 bp). Any deviation from this size (suggesting non-specific binding) was not used for analysis. A Qubit assay was used to determine concentration. Both measurements were then used to calculate the molar amounts for loading. FLASH software (Magoč and Salzberg, 2011), was used for assemblage of contiguous sequences and removal if found to be short after trimming for a base quality below 31. The uparse method (http://www.drive5.com/uparse/) was used to cluster contigs and remove chimera using version 7 of usearch (http://www.drive5.com/usearch/) (Edgar, 2013). Remaining contigs were assigned to operational taxonomic units (OTUs) via *de novo* OTU clustering with a 97% nucleotide identity. These OTUs were annotated using BLAST (Altschul et al., 1997) against the Greengenes (version 13_8) database (DeSantis et al., 2006). Principal component analysis (PCA) of ¼ root-transformed sequence data and α-diversity indices were performed using open access Past 3.13 software (Hammer et al., 2001), downloaded on May 12th, 2016. Using MetaboAnalyst 3.0 (Xia and Wishart, 2016) with a Euclidean distance measure and Ward clustering algorithm of cube root-transformed sequence data, heat maps were generated. Using the 16S rRNA amplicon dataset, the Phylogenetic Investigation of Communities by Reconstruction of Unobserved States (PICRUSt) software package (Langille et al., 2013) was used to predict functional capacity of fecal samples, and the HMP Unified Metabolic Analysis Network (HUMAnN) software package (Abubucker et al., 2012) was used to predict gene categories present at different levels between time points.

### Statistical analysis

For all samples, a cut-off of 10,000 high-quality reads (base quality >31) was set as inclusion for further analysis. Differences in α-diversity (average detected OTUs and Simpson Diversity Index) between pre-shipment and arrival samples were tested via *t*-test or Mann-Whitney rank sum test, depending on the normality of data. Differences in α-diversity between pre-shipment samples and samples from both facilities at 9 weeks were tested via ANOVA or Kruskal-Wallis ANOVA on ranks, depending on normality of data as pre-determined via Shapiro-Wilk normality testing. SigmaPlot 12.3 (Systat Software Inc., San Jose, CA) was used for all testing on the aforementioned data. Differences in β-diversity were tested using one-way permutational ANOVA (PERMANOVA) of ranked Bray-Curtis and Jaccard distances using Past 3.13 software (Hammer et al., 2001). Uncorrected *p*-values below 0.05 were considered significant and *p*-values between 0.05 and 0.07 were considered trends.

## Results

### Shipping

**Figure 2:**
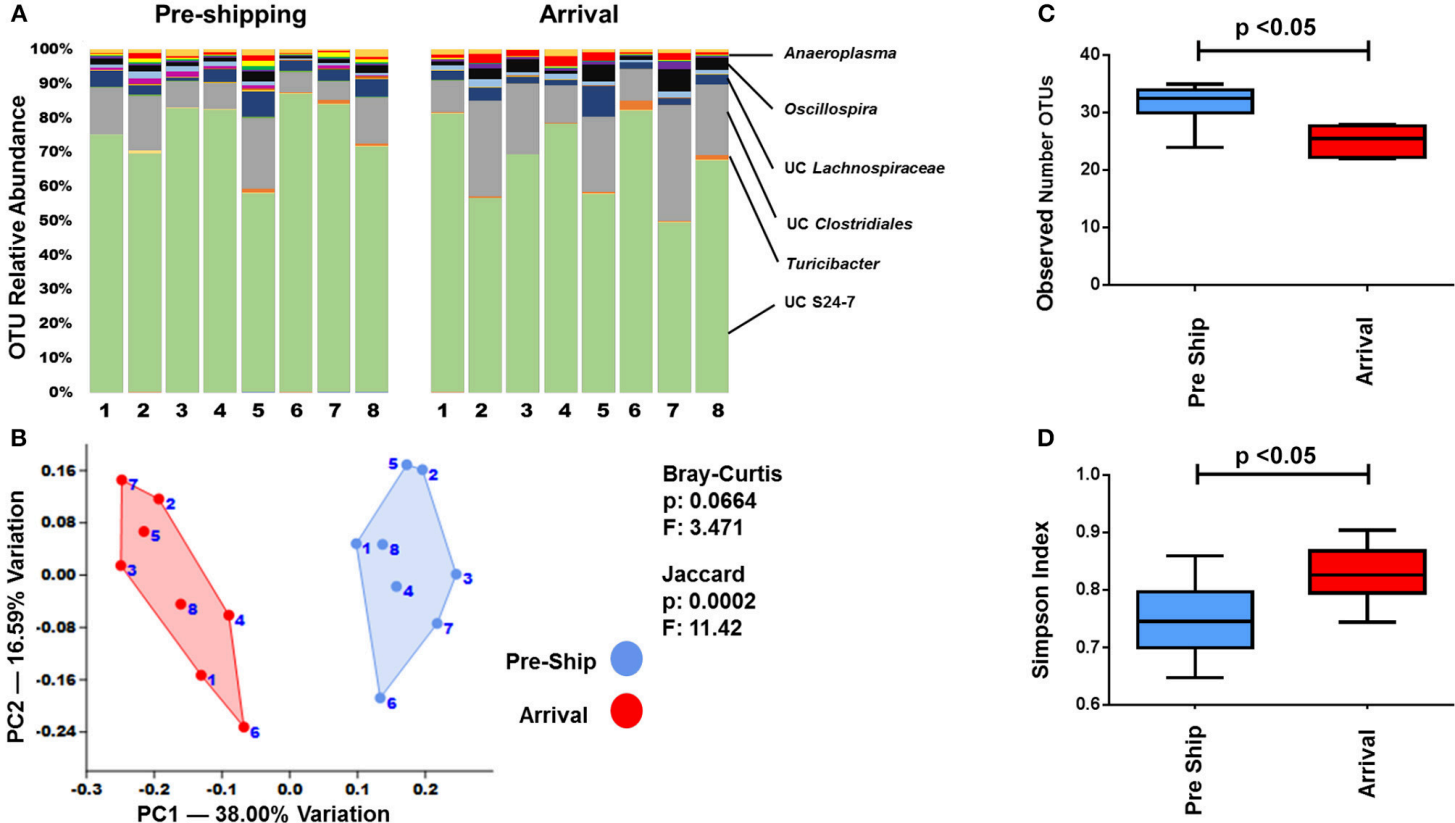
Transportation effects on the GM. **(A)** Bar charts showing the bacterial composition of adult B6J CON mice before and after shipping. Each bar represents an individual mouse and the relative abundance of OTUs observed in each are shown on the y-axis. Prominent families are labeled on the right. A complete taxonomic legend is provided in Supplemental Table 1. **(B)** PCA with Jaccard and Bray-Curtis PERMANOVA used to demonstrate differences in β-diversity. Numbers on figure correlate with numbers below bar chart. **(C,D)** Tukey box plots of α-diversity were used to further characterize changes pre and post shipping. Bars denote significant differences (*p* < 0.05) in mice.

In order to examine how a multi-day transportation event influenced the GM of mice, fecal samples from mice were collected immediately prior to shipping and immediately upon arrival. Fecal samples obtained upon arrival differed in their microbial profile in comparison to the pre-shipment samples (Figure 2, Supplemental Figures 1–4). Changes in the relative abundance of OTUs (groups of sequence sharing 97% nucleotide identity) between pre-shipment and arrival samples were apparent (Figure 2A, Supplemental Figure 1). Many of these changes were most identifiable in the less abundant OTU genera such as unclassified (UC) *Peptostreptococcaceae*, UC *Erysipelotrichaceae*, UC *Anaeroplasma*, UC *Turicibacter*, and UC *Mogibacteriaceae:* more dominant OTUs, such as UC *Clostridiales* and S24-7, were less susceptible to the initial effects of shipping.

To better assess the overall relatedness and differences between the bacterial communities immediately prior to and after shipping, principal component analyses (PCAs) were performed. PCAs collectively account for all of the detected OTUs, and by using dimension reduction, visually represent the degree of similarity and variance between samples in a two-dimensional plot. When analyzed by PCA, a clear separation of the pre-shipping and arrival time points was seen in the adult B6J CON mice (Figure 2B) with principal component (PC) 1 accounting for 38% of the variation. PERMANOVA, a non-parametric multivariate analysis of variation, showed that the pre-shipping and arrival time points significantly differed (*p* < 0.05) when using the Jaccard distance matrix analysis, but not when using the Bray-Curtis distance matrix analysis (Figure 2B). As a Jaccard analysis only accounts for the presence/absence of OTUs and a Bray-Curtis analysis is more sensitive to changes in OTU abundance, this supports that the initial compositional changes in the fecal microbiota were more likely influenced by a loss or gain of species than by changes in abundance of present species. However, with a Bray-Curtis *p*-value of 0.0664, there is a trend toward changes in abundance of pre-existing taxa. These trends are supported by the richness and diversity analyses as well. There was a significant difference (*p* < 0.05) in the species richness between the pre-shipping and arrival time points, which was calculated using a direct measure of the average number of identified OTUs (Figure 2C). Richness decreased following shipment, indicating a significant loss of OTUs, or decrease in relative abundance to a level below the limit of detection, following transportation. There was also a significant difference (*p* < 0.05) in the Simpson diversity index (Figure 2D), which specifically accounts for species dominance or evenness (Magurran, 2013).

Although specific metrics in the PERMANOVA, richness, and diversity varied for each group, a similar pattern of change was observed in the PCA of mice in the other groups as well (Supplemental Figures 2–4). Specifically, while there was clear separation by PCA in the adult CJ CON mice, PERMANOVA did not demonstrate significant variation between pre-shipping and arrival fecal samples using both Jaccard and Bray-Curtis distance measurements. As the Jaccard *p*-value was 0.0509, there did appear to be a similar trend to that observed in the adult B6J CON mice (Supplemental Figure 2). With a slight overall decrease in the mean richness and increase in the mean Simpson diversity index (Supplemental Figures 3, 4) adult CJ CON mice also followed a similar pattern as seen in the adult B6J CON mice. In both the weanling B6J CON mice and adult B6J CON2 mice, there was a significant difference in PERMANOVA using the Bray-Curtis analysis, but not the Jaccard analysis (Supplemental Figure 2). The richness and diversity indices are again supportive of these findings, which showed a significant change in the Simpson diversity index but not in the species richness (Supplemental Figures 3, 4). While it is well established that the GM of adult and weanling of mice differ, which was also seen here, it is interesting to note that both had a similar pattern of change to their bacterial communities using these metrics.

Interestingly, the group of adult B6J BAR mice appeared to demonstrate the most resilience to the immediate effects of shipping as there was no clear separation by PCA, no significant differences by PERMANOVA, and no significant changes to the richness and diversity (Supplemental Figures 2–4). Also, there was a slight difference in the pattern of change between the shipments of adult B6J CON and adult B6J CON2 mice shipped at different times (Figure 2, Supplemental Figures 1–4). It is difficult to assess if these variations between groups were related to differences in shipping (i.e., placement in truck, density of surrounding cages, lighting, seasonal variation, etc.) or differences in individual physiologic variations in response to a stressful situation. As a large degree of variation in other acute physiologic responses to shipping has been demonstrated (Landi et al., 1982; Aguila et al., 1988; van Ruiven et al., 1998; Swallow et al., 2005; Capdevila et al., 2007; Laroche et al., 2009; Shim et al., 2009; Arts et al., 2012; Lee et al., 2012), it is not surprising to see variations in the acute responses of the GM as well.

**Figure 3:**
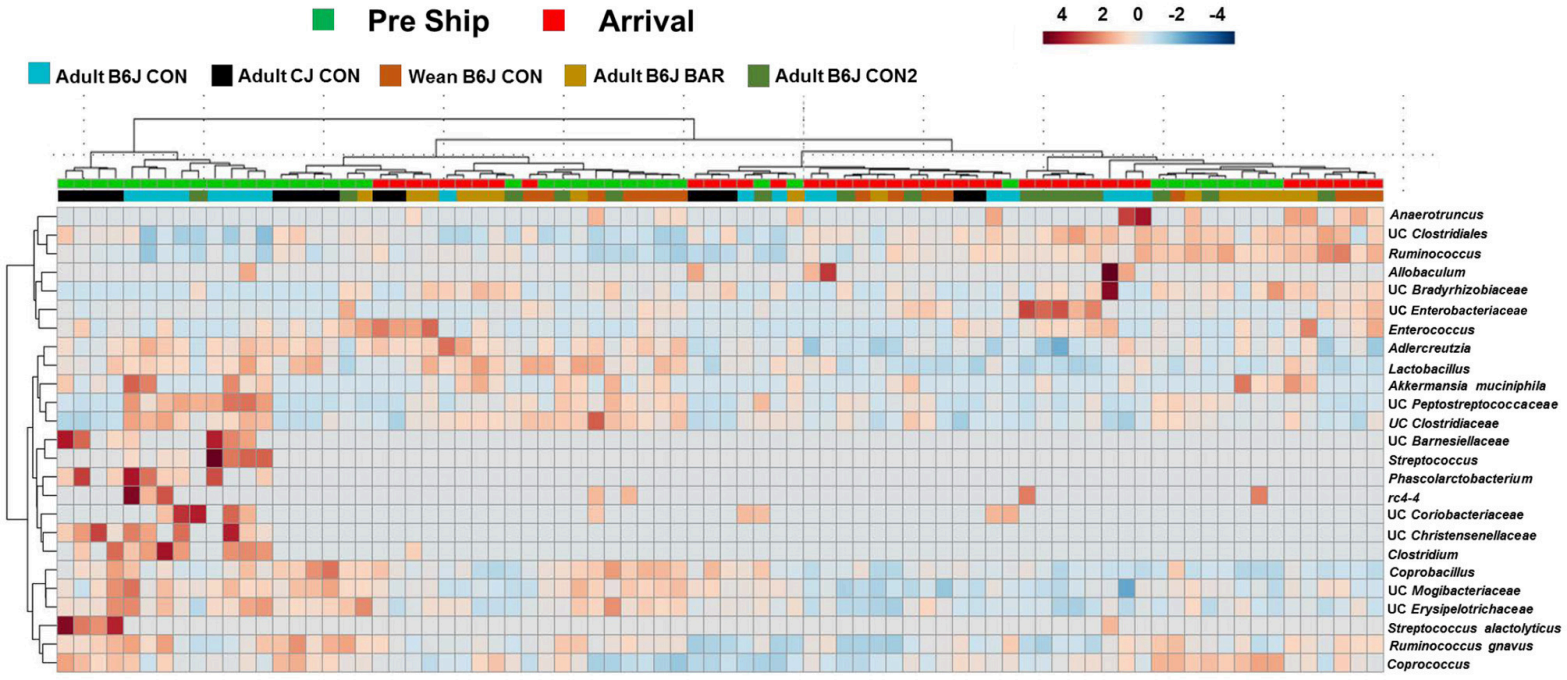
Hierarchical cluster analysis of all mice pre and post shipping. Hierarchical cluster analysis showing the top 25 OTUs with the most variation before shipping and upon arrival. Color intensity shows cube root transformed normalized OTU abundances in each sample. Colored bars on top denote pre-shipping samples from arrival samples. Colored bars along bottom denote the group the mice belonged to.

To better visualize specific OTUs that were susceptible to acute changes following shipping amongst all the groups, a heatmap was generated (Figure 3). This heatmap showed the 25 OTUs that demonstrated the greatest differences between pre-shipping and arrival. While there was no clear separation based on time point, the pattern suggests that some of the changes in these OTUs may occur consistently across strain, age, and housing status. For example, the abundance of many OTUs such as UC *Streptococcus*, UC *Clostridium*, and UC *Peptostreptococcaceae* were at greater relative abundance in many of the pre-shipping samples relative to the arrival samples, while OTUs such as UC *Clostridiales* were detected at a lower abundance in relation to the arrival samples. Conversely, OTUs like UC *Enterobacteriaceae* and UC *Anaerotruncus* were at greater relative abundance in arrival samples compared to pre-shipping samples while OTUs such as UC *Adlercreutzia* were at a lower abundance relative to pre-shipping. Despite the different groups having different starting and arrival GMs, these data identified certain genera of OTUs that may be most susceptible to the immediate effects of shipping regardless of strain, age, or housing status.

### Acclimation

**Figure 4:**
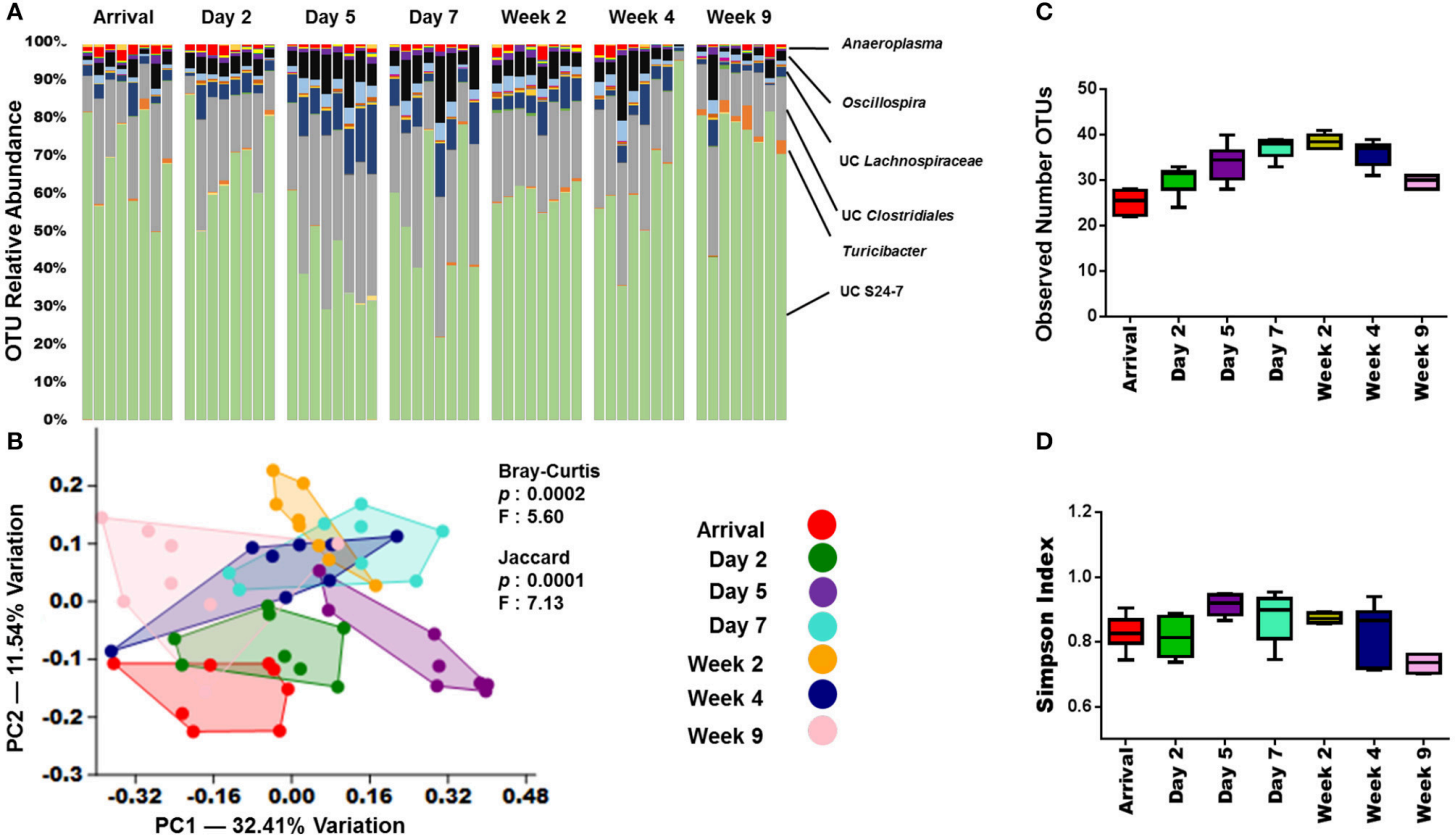
GM changes throughout acclimation.**(A)** Bar charts showing the bacterial composition of adult B6J CON mice from arrival through 9 weeks. Each bar represents an individual mouse and the relative abundance of OTUs observed in each are shown on the y-axis. Time points are labeled across the top of bars. Prominent families are labeled on the right. A complete taxonomic legend is provided in Supplemental Table 1. **(B)** PCA with Jaccard and Bray-Curtis PERMANOVA used to demonstrate differences in β-diversity. Legend of color coded time points is on the right. **(C,D)** Tukey box plots used to demonstrate differences in richness and diversity. One-way ANOVA demonstrated mice had significant overall differences (*p* < 0.05) in richness and diversity. Multiple comparison procedures (Student-Newman-Keuls method) revealed numerous significant differences between time points (not shown).

In an effort to understand how long the effects of shipping on the GM persist in the mouse, several more fecal samples from arrival through 9-weeks were collected. Visual examination of the OTU relative abundance and richness and diversity plots in adult B6J CON group suggested that the microbiota continues to change throughout acclimation (Figures 4A,C,D). As with the pre-shipping and arrival sampling data, these shifts appeared most dramatically in the less abundant OTUs. However, throughout the first week following arrival, there also appeared to be a shift in the more abundant OTUs. For example, the relative abundance of UC S24-7 decreased before increasing again around the day 7 time point. OTU abundances for other groups of mice show that exact changes may vary, but similar patterns can be observed, especially with the more rare OTUs appearing to undergo the most dramatic shifts in the first 5 days following arrival (Supplemental Figure 5). To better characterize the acclimation-associated changes in microbiota, PCA/PERMANOVA were again performed. These analyses indicated significant differences (*p* < 0.05) amongst the time points using both Jaccard and Bray-Curtis distances (Figure 4B). Interestingly, along with the arrival time points, the day 2 and day 5 time points stood out from the rest of the time points along PC 2 (Figure 4B). This was supported by a pairwise comparison of time points (Supplemental Table 2) which demonstrated that the day 5 time point differed significantly (*p* < 0.05) from all other time points except day 7 using the analysis of Bray-Curtis distances. Also of note, is that in day 7 and beyond, the time points did not differ significantly with the exception of week 9. A similar pattern was seen in the other groups of mice with the day 2 and/or day 5 post-arrival time point shifted away from the other time points in 4/5 groups of mice analyzed (Figure 4B, Supplemental Figure 6). Interestingly, the adult B6J CON2 mice did not share this pattern, with only the arrival time point showing separation (Supplemental Figure 6). Pairwise comparisons of the other groups of mice showed similar patterns with the analysis of Bray-Curtis distances showing far fewer significant changes that the analysis of Jaccard distances (Supplemental Table 4). Also similar was that when examining the Bray-Curtis distances, the GM appeared to stabilize around day 7 in the adult CJ CON and weanling B6J CON mice. The adult B6J BAR and adult B6J CON2 mice showed the most stable (no significant differences when analyzing α-diversity, β-diversity and/or no clear separation on PCA) GM throughout acclimation with little significant changes in the analysis of Bray-Curtis distances. When comparing the pairwise Jaccard distances between groups, there was much variation between groups with the adult CJ CON group showing no stabilization and the B6J CON2 group showing stabilization from day 2 through week 4 (Supplemental Table 4).

**Figure 5:**
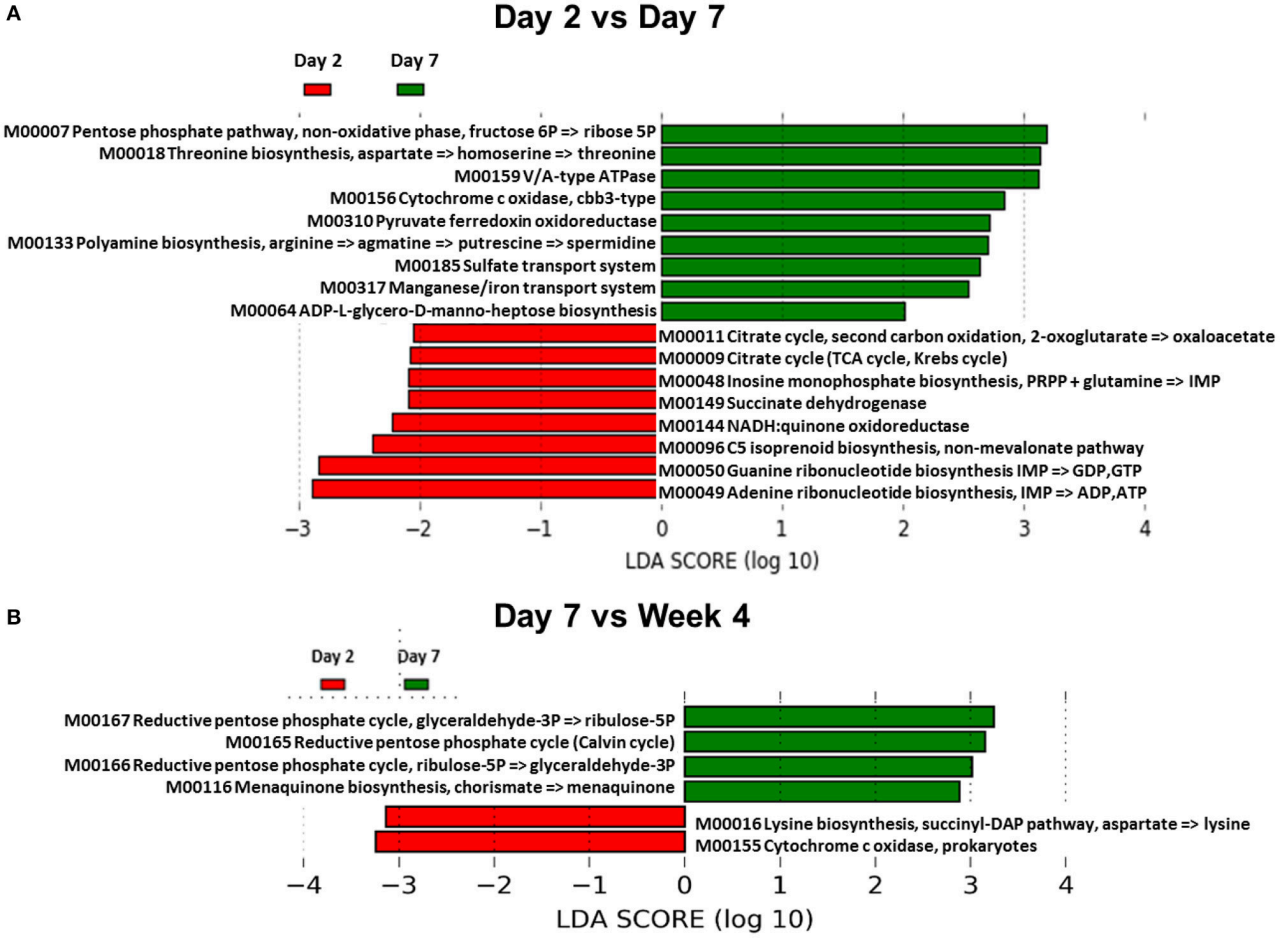
Functional changes of the GM during acclimation. **(A)** Linear discriminant analysis effect size (LEfSe) generated from PICRUSt and HUMAnN data showing differences in predicted metabolic function of gut bacteria between day 2 and day 7 in adult B6J CON mice. Green bars indicate pathways that are upregulated in day 7 compared to day 2 and red bars indicate pathways that are upregulated in day 2 compared to day 7. An LDA score of 2 indicates significance of *p* < 0.05. **(B)** LEfSe data showing functional differences between day 7 and week 4.

We also sought to determine whether differences in the composition of the GM also resulted in changes in GM-associated metabolic function. In order to accomplish this, Phylogenetic Investigation of Communities by Reconstruction of Unobserved States (PICRUSt) and Human Microbiome Project Unified Metabolic Analysis Network (HUMAnN) software packages were used. PICRUSt is able to predict the metabolic functional capacity of microbial communities based on the gene content of previously sequenced bacterial genomes. HUMAnN then applies a non-parametric Kruskal-Wallis test to the predicted data to determine the effect size in each group of samples. This is then visualized using a supervised linear discriminant analysis. Using this approach, the GM was analyzed comparing day 2 vs. day 7, day 7 vs. week 4, and day 7 vs. week 9 (Figure 5). There were several predicted functional differences in pathways when comparing the day 2 and day 7 time points (Figure 5A). From day 7 to week 4 there were relatively few predicted functional differences between the GMs (Figure 5B), however, from day 7 to week 9 there were again several predicted functional differences (Supplemental Figure 7). This indicated that the changes in abundances observed between days 2 and 7 are also predicted to lead to functional differences in the GM. It also showed that the GM appears to be relatively stable with regard to function, between day 7 and week 4. Interestingly, although a large separation between day 7 and week 9 was not observed by PCA (Figure 4B), there appeared to be numerous predicted functional differences between the two time points. These functional metabolic predictions did align with the PERMANOVA pairwise analysis, which demonstrated significant differences between day 2 and 7, and between day 7 and week 9, but not between day 7 and week 4 (Supplemental Table 2).

Taken together, these results showed that while the acclimation varied for each group of mice, there did appear to be a pattern of significant changes to the GM happening within the first 5 days following arrival. As noted before, minor differences in the shipping process and different individual responses to stress may account for the variation observed.

### Institutionalization

**Figure 6:**
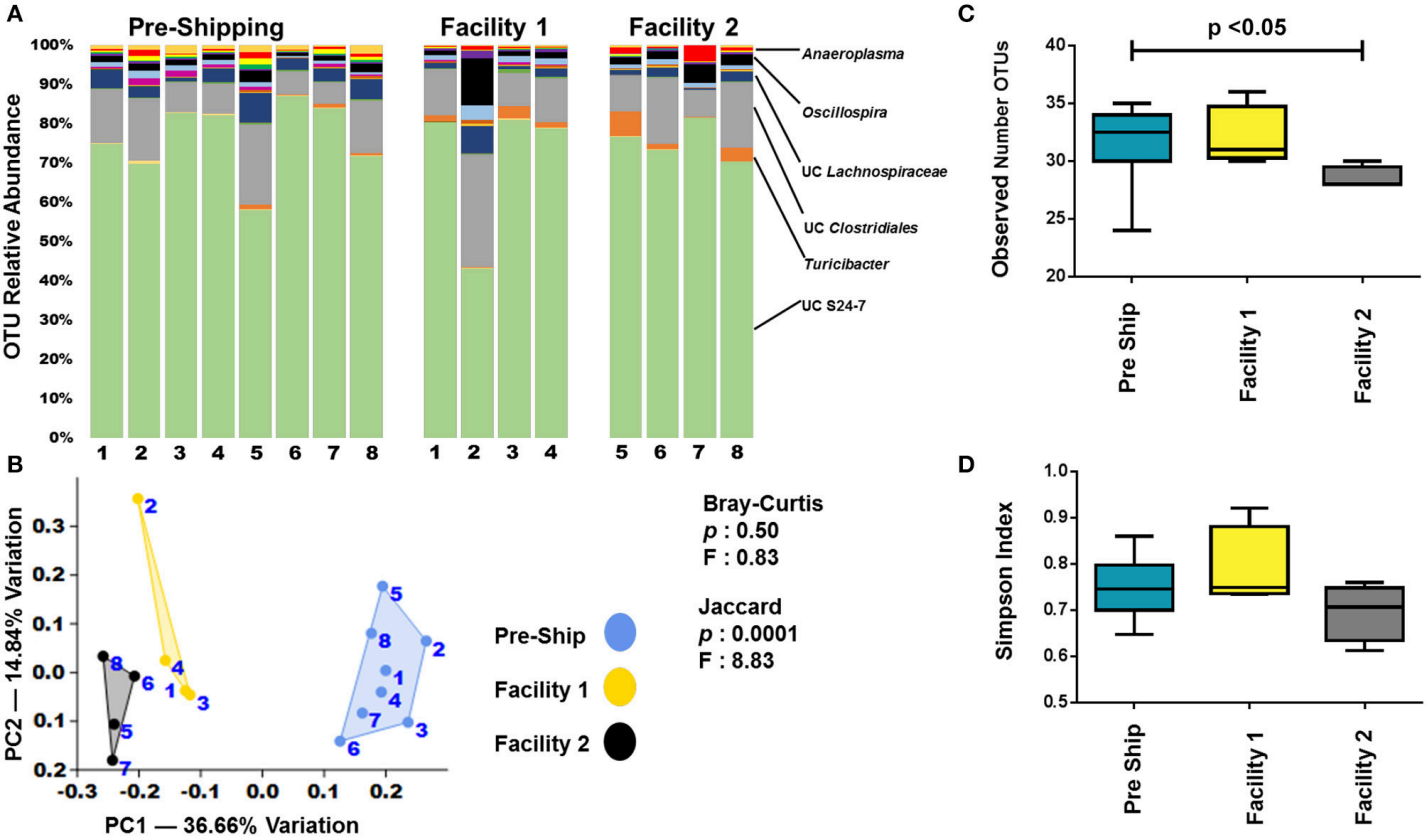
Institutionalization of the GM. **(A)** Bar charts comparing the bacterial composition of adult B6J CON mice prior to shipping and after 9 weeks in two facilities at the arrival institution. Each bar represents an individual mouse and the relative abundance of OTUs observed in each are shown on the y-axis. Prominent families are labeled on the right. A complete taxonomic legend is provided in Supplemental Table 1. **(B)** PCA with Jaccard and Bray-Curtis PERMANOVA showing differences between institutions as well facilities. Numbers on figure correlate with numbers below bar chart. **(C,D)** Tukey box plots of α-diversity were used to further characterize changes between institutions and facilities. Bars denote significant differences (*p* < 0.05) in mice.

In order to determine if mice developed a GM profile unique to our institution following transportation and acclimation, fecal samples from the pre-shipment time points were compared to the final week 9 time point. Additionally, we speculated that separate facilities within an institution could lead to GM drift, but that this could be minimized if housing conditions were kept identical. To determine this, each group of mice was divided and housed under identical conditions at two separate facilities. As with pre-shipment and arrival samples, the largest changes in relative abundance of OTUs between pre-shipment and facilities, as well as between facilities themselves, was observed in less abundant OTUs (Figure 6A). It was also apparent when examining the relative abundances of OTUs, that there were greater differences between the pre-shipment samples and the facilities than there were differences between the facilities themselves (Figure 6A). Further examination by PCA of the final week 9 time-point revealed that the GM never completely reverted to the GM seen at The Jackson Laboratory (Figure 6B). Additionally, it showed separation based on the facility in which they were housed following arrival (Figure 6B). Of note and in agreement with the observed differences in relative abundance, the separation along PC1 between facilities was much smaller than the separation between the pre-shipment samples and each of the facilities themselves, indicating the change that occurred between the facilities was less than the change that occurred between the initial and receiving institutions. A similar pattern of separation was observed in other groups of mice examined as well (Supplemental Figures 8–11). PERMANOVA comparing pre-shipment from each of the facilities at the week 9 time-point found an overall significance (*p* < 0.05) using the analysis of Jaccard distances, but not using the analysis of Bray-Curtis distances (Figure 6B). The significance in the Jaccard distance measurements can likely be explained by a loss of OTUs, with a significant (*p* < 0.05) decrease in OTUs seen between the pre-shipment sample and facility 2 (Figure 6C). In agreement with the Bray-Curtis distances measurements, no significant changes in diversity was detected between pre-shipment samples and facilities (Figure 6D). A pairwise comparison of the pre shipment samples and facilities at 9 weeks revealed significant differences (*p* < 0.05) between pre-shipment samples and each facility using the analysis of Jaccard distances, but not the analysis of Bray-Curtis distances (Supplemental Table 3). PERMANOVA of the adult B6J BAR group demonstrated a similar pattern of significance, while all other groups showed significance using both Jaccard and Bray-Curtis distance measurements (Supplemental Figure 9). Pairwise comparisons of other groups of mice varied in their significance, but supported the idea that greater changes happened between institutions than between the facilities (Supplemental Table 5). As there was a multi-day transportation event that occurred between institutions, as well as changes in diet, bedding, caging, care-staff, etc. (none of which occurred between the facilities), it is not surprising that greater GM drift was observed between institutions than between facilities.

**Figure 7:**
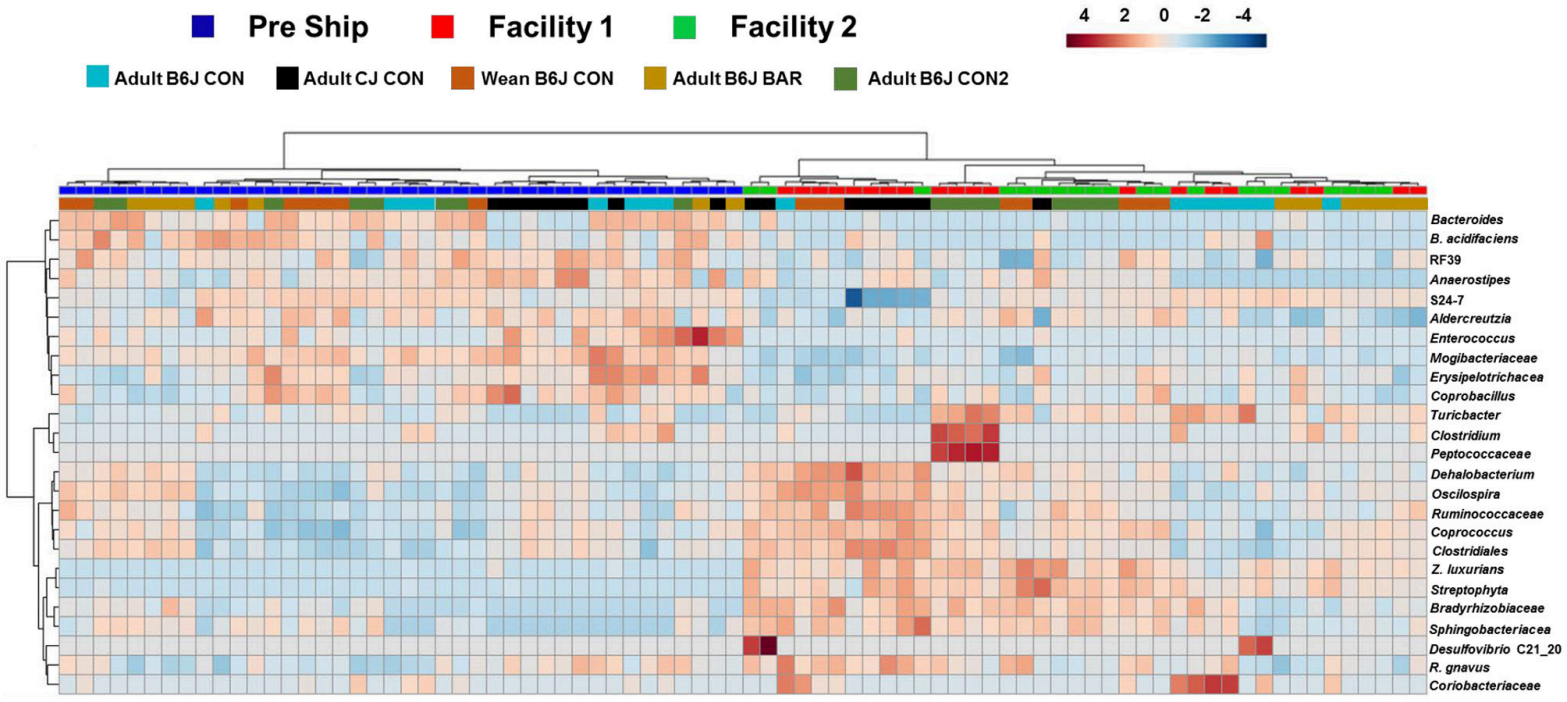
Hierarchical cluster analysis of all mice prior to shipping and following 9 weeks at new facilities. Hierarchical cluster analysis showing the top 25 OTUs with the most variation between our facilities and The Jackson Laboratory. Color intensity shows cube root transformed normalized OTU abundances in each sample. Colored bars on top denote pre-shipping samples from samples at each facility following 9 weeks. Colored bars along bottom denote the group the mice belonged to.

A heatmap of the OTU abundance data for these time points was supportive of the previous conclusions (Figure 7). When the top 25 OTUs that had the largest changes in abundance were examined, there was complete separation of the mice based on the pre-shipment and week 9 time points, and, while there was a pattern of separation based on the facility in which they were housed, this delineation was much less clear. Specifically, many of the OTU abundances in the upper left panel, such as *Bacteroides acidifaciens* were detected at a greater relative abundance at The Jackson Laboratory in comparison to those seen at our institution following 9 weeks. Here we also see that even some of the major GM taxa, such as UC S24-7, showed differing abundances between institutions.

Where the heatmap demonstrating acute changes in shipping had a less clear delineation between shipping and arrival, this data (representing a longer more chronic change) showed a much clearer separation between institutions. This data provides a framework of OTU genera that may be most susceptible to long term drift of the murine GM at an institution.

## Discussion

Here we show that the GM of mice is altered by the process of transportation from one institution to another. As mentioned, environmental changes and stressors can cause alterations in the GM of mice. Because the process of going through a multi-day shipment may include variations in housing, lighting, chow, etc., it was not surprising to find that an extended transportation event could also induce changes in the GM of mice. Aside from the effects that stress may play on the GM, one can speculate that mice having access to a more simplified and decreased fiber diet in the form of gel packs during the shipping process may not support some of the rarer bacteria. While these bacteria may not have been detected upon arrival, they still may have been occupying small niches in the gut at levels below detection and subsequently could potentially repopulate given time. These decreases in rare OTUs likely lead to a greater evenness of remaining OTUs, hence the increase seen in the Simpson diversity index that frequently accompanied decreases in richness of our mice. The opposite of this could happen with bacteria that were not detected prior to shipping, but thrive in the context of a gel diet. While the specific changes in richness and diversity varied for each group, certain OTUs did stand out in the heatmap that suggest they may be the most susceptible to change with shipping regardless of age, strain, or housing source.

This work contributes to the growing body of data highlighting the impacts of prolonged transportation in mice and the need for determining appropriate acclimation periods (Landi et al., 1982; Aguila et al., 1988; Ruiven et al., 1996; van Ruiven et al., 1998; Swallow et al., 2005; Conour et al., 2006; Obernier and Baldwin, 2006; Capdevila et al., 2007; Laroche et al., 2009; Shim et al., 2009; Arts et al., 2012; Lee et al., 2012). It should be noted that this study is somewhat limited in that it only examined mice shipped from The Jackson Laboratory, and as many investigators are aware, the shipping methods and containers used to house the mice during shipping can vary greatly between different repositories or institutions. As there were also differences in the pattern of change between the adult B6J CON and adult B6J CON2 mice shipped at different time points, it supports that there may be differences in shipping environment or seasonality that can affect the GM. Differences in the GM of mice following separate shipments have been reported (Hoy et al., 2015) previously. In addition to differing environmental conditions during shipment, these differences could also be a result of differing social groups and maternal genetics and transfer of the GM. An attempt was made to minimize these latter differences by only using mice reared within the same room at The Jackson Laboratory. While the precise environmental and husbandry conditions between shipments were not controlled in this study, we did not feel it necessary as there could always be minor variations in cage placement, temperature, lighting, noise, seasonal changes, etc. that could never practically be kept identical for all shipments. Also, of importance is the time that mice spend in transit based on geographic location. In this case, the mice spent 5 days in the transportation process, but this time could be increased or decreased depending on proximity of the institution to the repository, or on weather conditions during travel that may necessitate slower driving or delays.

Based on past studies, several recommendations have been made regarding the acclimation period needed for mice following transportation from a repository or other research institution (Ruiven et al., 1996; Swallow et al., 2005; Conour et al., 2006; Obernier and Baldwin, 2006; Capdevila et al., 2007). Because of this research, many institutions have a required minimum of 2 days acclimation to a new facility following arrival. Here we show that the significant changes in the GM of mice often occur at 5 days post-arrival, which supports the idea that investigators, specifically those analyzing the GM and its potential role in health and disease states, may need to extend this acclimation period longer than 2 days. This is in slight contrast to other studies that have examined changes in the GM of mice following transportation of mice within a facility where it was reported that stabilization occurred at 5 days (Ma et al., 2012). Likely, the extended length of shipping and changes in husbandry in this study gave rise to more significant alterations in the GM requiring a slightly longer period of acclimation. The most stable period of the GM for the mice in this study appeared to occur between day 7 and week 4 based on both OTU abundances and predicted functional differences. This is valuable information in that many acute studies could occur during this time, and would likely be done under a relatively stable GM. Of note however, were the changes in the GM noted at 9 weeks following arrival. As no significant alterations in the housing or environment for either facility occurred at this week 9 time point, we can surmise that the observed shifts in the GM were a normal process. These periodic shifts in the GM of rodents have been reported (Thompson et al., 2010; Hoy et al., 2015) and while not fully understood, some research has shown the normal aging process can contribute to changes in the GM of mice (Langille et al., 2014). For investigators specifically concerned with changes in the GM of their mice, this highlights the need for continual monitoring of control mice to be aware of such shifts that may occur.

Another phenomenon noted in laboratory mice has been the differences in the GM of genetically similar mice housed at different mouse repositories or vendors. Past studies demonstrated that mice of similar genetic background obtained from different repositories harbor GM profiles unique to their source of origin (Ericsson et al., 2015). In addition to this, we now understand that institutions can also harbor unique GM profiles distinct from the repositories in which mice were obtained (Rausch et al., 2016). However, we are unware of any studies that have documented how quickly this drift at an institution can occur following arrival of mice from a repository. Here we show that following as little as 9 weeks, the mice harbored a GM profile distinct from that seen at The Jackson Laboratory. We have also identified a list of potential taxa that, regardless of strain, age, and prior housing, appeared most susceptible to this drift. Understanding that specific housing conditions can vary greatly across institutions, these identified OTUs could likely be different depending on the husbandry conditions at individual institutions. It should be noted that mice shipped to customers from The Jackson Laboratory Repository are all Specific Pathogen Free (SPF) re-derived animals maintained in barrier facilities, which minimizes the variability of GM and protects recipient institutions from introducing unwanted flora, also helping to promote reproducibility.

Furthermore, while there has also been work identifying GM variation based on facilities within institutions (Rausch et al., 2016), husbandry standards were not replicated between facilities to determine if this variation could be minimized. Here we have demonstrated that with strict control over husbandry practices, the GM drift between facilities can be minimal in comparison to the drift seen between institutions where husbandry practices were not controlled. As the husbandry was strictly controlled between facilities, we can only speculate that the minor variations in room environment such as noise, lighting, temperature, cage placement on racks, and the other mice occupying the room contributed to the observed separation in PCA between facilities. These data are important in demonstrating that GM shifts can be kept to a minimum between facilities when husbandry factors are also controlled. Had we made a greater attempt to keep husbandry and housing (caging, bedding, diet, water, etc.) identical to that seen at The Jackson Laboratory, we may have seen less of a shift in the GM between institutions as well. However, as we realized this would not likely occur or be possible for most institutions, we elected to use the standard husbandry practices used by our institution upon receiving the mice.

This study does have limitations. In an effort to characterize several different factors (age, strain, housing status, time of year) many comparisons of groups were made only once with a limited number of mice. Because many of the individual groups had different GMs to begin with, and the changes to specific OTUs were often unique to each group, it does make it more difficult to infer if these specific OTU changes can be applied to mice of other ages, strains, etc. However, we feel that taken as whole, the number of mice is quite large and while each group may have had unique changes to specific OTUs, the overall pattern of change and acclimation time was similar amongst many of the groups. Additionally, the study was limited by the predictive capabilities of using PICRUST to examine if the changes in the GM would lead to functional changes. Ideally, shotgun metagenomic sequencing could have given a more definitive answer to the functional changes, but this was not financially feasible for this study. Finally, as mentioned previously, this study only examined female mice due to complications arising from group housing and cage fighting amongst males. It is unclear if acclimation for male mice would follow a similar pattern and whether or not this pattern would be influenced by sexual differences as well as differences in single vs. group housing.

In conclusion, we have shown that the GM of mice is altered when mice are transported between institutions. While the acute changes varied between groups, specific bacteria were identified that appeared to consistently change following shipping regardless of strain, age, or prior housing status providing a potential list of acute transportation-sensitive OTUs. These alterations appeared to stabilize around 1 week following arrival, but this was variable, and as evidenced by the final time point, continued to undergo periodic shifts throughout the animals' lifetime. Additionally, we have shown that mice develop a GM distinctly different from the institution from which they originated following transportation and have identified specific OTUs at our institution that may be most susceptible to alterations in abundance over time. GM changes between facilities within an institution was minimal when housing and husbandry were kept similar. While more study is needed to determine if and how these alterations could lead to phenotypic changes in murine models of disease, this work contributes to the growing body of evidence showing that environmental and husbandry changes can have significant impacts on the GM of our laboratory mice.

## Author contributions

DM, AE, CL, and CF: conceived and designed the experiment; AE and CF: provided reagents and materials; KW: coordinated mice selection and shipping; DM: interpreted the data and wrote the manuscript; AE, CL, SB, KW, and CF: helped analyze data and review the manuscript.

### Conflict of interest statement

The authors declare that the research was conducted in the absence of any commercial or financial relationships that could be construed as a potential conflict of interest.
