# Supplementary material for: Acclimation and Institutionalization of the Mouse Microbiota Following Transportation
Source: Front Microbiol. 2018 May 28;9:1085. doi: 10.3389/fmicb.2018.01085 (PMC5985407; doi:10.3389/fmicb.2018.01085)
Supplement: Supplementary file 7 [file Image_7.pdf]

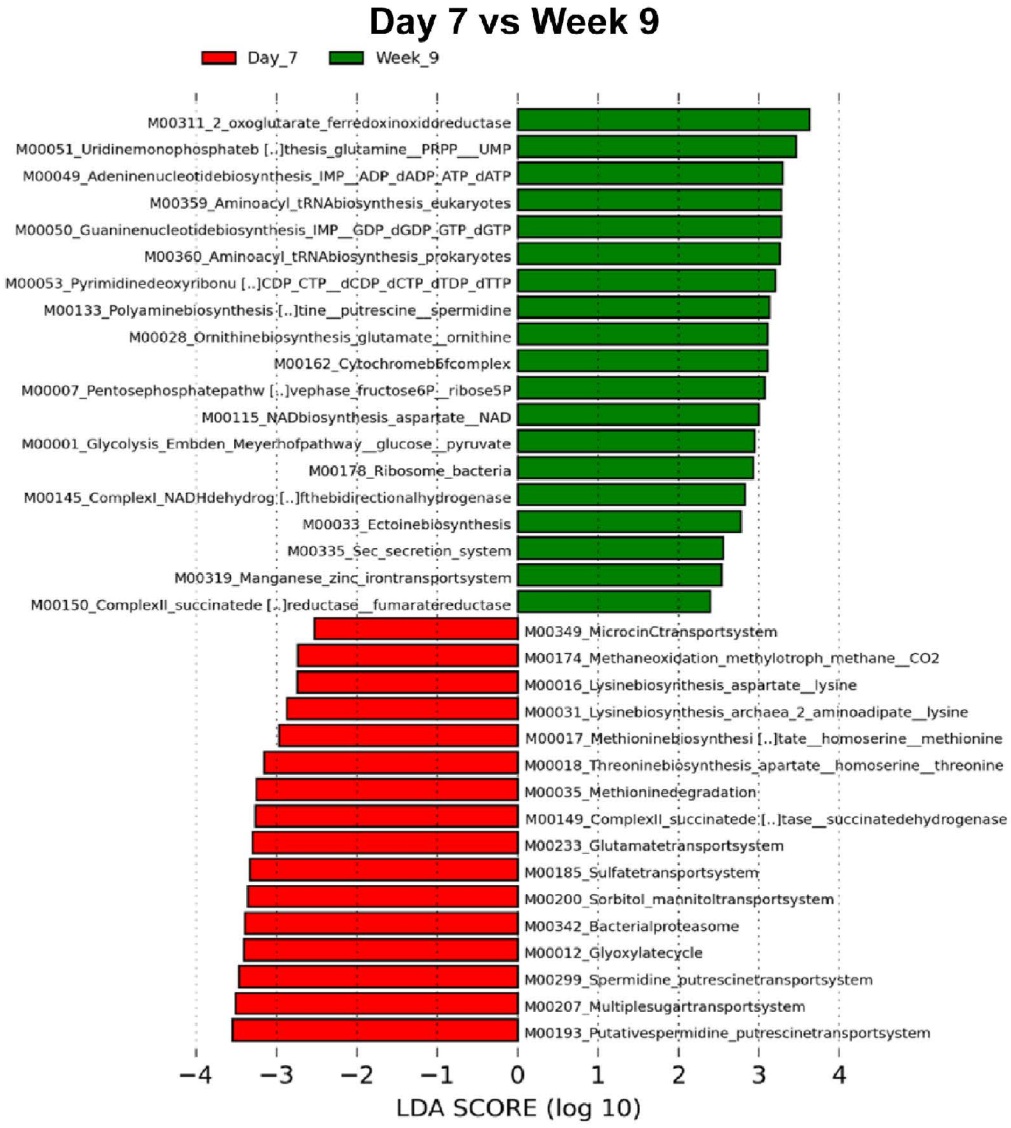

Supplemental Figure 7. Functional changes of the GM during acclimation. LEfSe data showing functional differences between day 7 and week 9.
