# Supplementary material for: Acclimation and Institutionalization of the Mouse Microbiota Following Transportation
Source: Front Microbiol. 2018 May 28;9:1085. doi: 10.3389/fmicb.2018.01085 (PMC5985407; doi:10.3389/fmicb.2018.01085)
Supplement: Supplementary file 12 [file Table_1.PDF]

Table 1

- Key for all OTUs

Methanobrevibacter	No blast hit	Microbacteriaceae	Microbacterium	Acidimicrobiales	Microthrixaceae
Coriobacteriaceae	Adlercreutzia	Bacteroidales	BF311	Bacteroides	Bacteroides acidifaciens
Prevotella	RF16	Rikenellaceae	AF12	Rikenella	S24-7
Chitinophagaceae	YS2	Streptophyta	Mucispirillum schaedleri	Bacillales	Fibrobacter succinogenes
Gemellaceae	Lactobacillales	Enterococcus	Lactobacillus	Lactobacillus mucosae	Streptococcus
Clostridium	SMB53	Dehalobacterium	EtOH8	Lachnospiraceae	Anaerostipes
Roseburia	Ruminococcus gnavus	Peptococcaceae	rc4-4	Peptostreptococcaceae	Peptostreptococcus
Phascolarctobacterium	Syntrophomonas	Syntrophomonas wolfei	Veillonellaceae	Megamonas	Mogibacteriaceae
Cetobacterium somerae	Alphaproteobacteria	Eubacterium biforme	SHA-1	RF32	Ellin5290
Methylobacterium	Agrobacterium	Carludovica palmata	Phytophthora ramorum	Zea luxurians	Sphingomonadaceae
Desulfovibrionaceae	Bilophila	Desulfovibrio C21_c20	Helicobacteraceae	Ralstonia	Desulfovibrio
Acinetobacter rhizosphaerae	Pseudomonadaceae	Moraxella	Pseudomonas	Stenotrophomonas	Pseudomonas viridiflava
Acholeplasmataceae	Acholeplasma	Anaeroplasma	RF39	ML615J-28	RFP12
Methanoculleus	MVS-40	Pseudoclavibacter helvolus	Actinoplanes	Propionibacterium acnes	Pseudonocardia halophobica
Marinilabiaceae	Porphyromonadaceae	Parabacteroides	Parabacteroides distasonis	Prevotellaceae	Porphyromonas
Barnesiellaceae	Odoribacter	Paraprevotella	Sphingobacteriaceae	Sediminibacterium	T78
TSCOR003-O20	Paenibacillaceae	Geobacillus	Bacillus	Bacillus flexus	Staphylococcus succinus
Streptococcus alactolyticus	Turicibacter	Clostridiales	Christensenellaceae	Caldicoprobacter	Clostridiaceae
Blautia	Blautia producta	Butyrivibrio	Coprococcus	Dorea	Lachnospira
Ruminococcaceae	Anaerotruncus	Faecalibacterium prausnitzii	Oscillospira	Ruminococcus	Ruminococcus flavefaciens
Parvimonas	Thermoanaerobacterales	Erysipelotrichaceae	Allobaculum	Catenibacterium	Coprobacillus
Rhizobiales	Bradyrhizobiaceae	Devosia	Ochrobactrum intermedium	Hyphomicrobium	Paracoccus
Sphingomonas	Betaproteobacteria	Sphingopyxis alaskensis	Sutterella	Comamonadaceae	Delftia
Helicobacter	Shewanella	Aeromonadaceae	Enterobacteriaceae	Pasteurellaceae	Acinetobacter
Xanthomonadaceae	Xanthomonas	Treponema	HA73	F16	Candidatus Phytoplasma
Akkermansia muciniphila					

Supplemental Table 1. Legend of all OTUs. Each color denotes OTUs for all bar charts in primary and supplemental figures.
