# Supplementary material for: Acclimation and Institutionalization of the Mouse Microbiota Following Transportation
Source: Front Microbiol. 2018 May 28;9:1085. doi: 10.3389/fmicb.2018.01085 (PMC5985407; doi:10.3389/fmicb.2018.01085)
Supplement: Supplementary file 13 [file Table_2.pdf]

(A) Bray-Curtis Index

	Arrival	Day 2	Day 5	Day 7	Week 2	Week 4	Week9
Arrival		0.637	8E-04	0.045	0.04	0.3376	0.2898
Day 2	0.637		6E-04	0.047	0.0628	0.3725	0.2442
Day 5	8E-04	6E-04		0.175	0.0007	0.01	0.0007
Day 7	0.045	0.047	0.175		0.1517	0.2561	0.015
Week 2	0.04	0.063	7E-04	0.152		0.5632	0.0093
Week 4	0.338	0.373	0.01	0.256	0.5632		0.1038
Week 9	0.29	0.244	7E-04	0.015	0.0093	0.1038	

(B) Jaccard Index

	Arrival	Day 2	Day 5	Day 7	Week 2	Week 4	Week 9
Arrival		4E-04	4E-04	1E-04	0.0002	0.0003	0.0004
Day 2	4E-04		0.005	3E-04	0.0001	0.0003	0.0005
Day 5	4E-04	0.005		1E-04	0.0001	0.0005	0.0003
Day 7	1E-04	3E-04	1E-04		0.8486	0.0334	0.0001
Week 2	2E-04	1E-04	1E-04	0.849		0.7896	0.0001
Week 4	3E-04	3E-04	5E-04	0.033	0.7896		0.0003
Week 9	4E-04	5E-04	3E-04	1E-04	0.0001	0.0003	

Supplemental Table 2. Pairwise tables comparing time points during acclimation. (A) Bray-Curtis and (B) Jaccard index pairwise tables comparing arrival through week 9 time points. Boxes in red indicate significant ($p<0.05$) differences between time points.
