# Supplementary material for: Acclimation and Institutionalization of the Mouse Microbiota Following Transportation
Source: Front Microbiol. 2018 May 28;9:1085. doi: 10.3389/fmicb.2018.01085 (PMC5985407; doi:10.3389/fmicb.2018.01085)
Supplement: Supplementary file 14 [file Table_3.pdf]

(A) **Bray-Curtis Pairwise**

	Pre-Ship	Facility 1	Facility 2
Pre-Ship		0.3764	0.3181
Facility 1	0.3764		0.8641
Facility 2	0.3181	0.8641	

(B) **Jaccard Pairwise**

	Pre-Ship	Facility 1	Facility 2
Pre-Ship		0.0026	0.002
Facility 1	0.0026		0.0267
Facility 2	0.002	0.0267	

Supplemental Table 3. Pairwise tables of institutionalization. (A) Bray-Curtis and (B) Jaccard index pairwise tables comparing pre-shipping samples to each facility at nine weeks. Boxes in red indicate significant ($p<0.05$) differences between time points.
