# Supplementary material for: Acclimation and Institutionalization of the Mouse Microbiota Following Transportation
Source: Front Microbiol. 2018 May 28;9:1085. doi: 10.3389/fmicb.2018.01085 (PMC5985407; doi:10.3389/fmicb.2018.01085)
Supplement: Supplementary file 15 [file Table_4.PDF]

(A) Bray-Curtis Index

(B) Jaccard Index

Adult CJ CON

(A) Bray-Curtis Index

	Arrival	Day 2	Day 5	Day 7	Week 2	Week 4	Week9
Arrival		0.138	0.005	2E-04	0.0005	0.0183	0.0022
Day 2	0.138		0.059	2E-04	0.001	0.1095	0.0106
Day 5	0.005	0.059		0.003	0.0831	0.3691	0.1009
Day 7	2E-04	2E-04	0.003		0.226	0.0062	0.3206
Week 2	5E-04	0.001	0.083	0.226		0.0692	0.4113
Week 4	0.018	0.11	0.369	0.006	0.0692		0.1425
Week 9	0.002	0.011	0.101	0.321	0.4113	0.1425	

(B) Jaccard Index

	Arrival	Day 2	Day 5	Day 7	Week 2	Week 4	Week9
Arrival		5E-04	5E-04	1E-04	0.0004	0.0001	0.0003
Day 2	0.0005		0.04	1E-04	0.0032	0.0011	0.0003
Day 5	0.0005	0.04		3E-04	0.0016	0.0002	0.0007
Day 7	0.0001	1E-04	3E-04		0.0009	0.0194	0.0002
Week 2	0.0004	0.003	0.002	9E-04		0.001	0.0003
Week 4	0.0001	0.001	2E-04	0.019	0.001		0.0001
Week 9	0.0003	3E-04	7E-04	2E-04	0.0003	0.0001	

Wean B6J CON

(A) Bray-Curtis Index

	Arrival	Day 2	Day 5	Day 7	Week 2	Week 4	Week9
Arrival		0.028	3E-04	0.023	0.1445	0.1584	0.1855
Day 2	0.028		0.027	0.384	0.1696	0.0477	0.216
Day 5	3E-04	0.027		0.002	0.0025	0.0008	0.0023
Day 7	0.023	0.384	0.002		0.2803	0.07	0.3031
Week 2	0.145	0.17	0.003	0.28		0.3907	0.7861
Week 4	0.158	0.048	8E-04	0.07	0.3907		0.3313
Week 9	0.186	0.216	0.002	0.303	0.7861	0.3313	

(B) Jaccard Index

	Arrival	Day 2	Day 5	Day 7	Week 2	Week 4	Week9
Arrival		1E-04	2E-04	1E-04	0.0003	0.0002	0.0005
Day 2	0.0001		0.735	6E-04	0.2211	0.2055	0.1007
Day 5	0.0002	0.735		0.004	0.0557	0.0287	0.0587
Day 7	0.0001	6E-04	0.004		0.0067	0.0011	0.0002
Week 2	0.0003	0.221	0.056	0.007		0.1779	0.0641
Week 4	0.0002	0.206	0.029	0.001	0.1779		0.0778
Week 9	0.0005	0.101	0.059	2E-04	0.0641	0.0778	

Adult B6J BAR

(A) Bray-Curtis Index

	Arrival	Day 2	Day 5	Day 7	Week 2	Week 4	Week9
Arrival		0.631	0.175	0.27	0.6593	0.1355	0.5481
Day 2	0.631		0.297	0.388	0.4156	0.2223	0.5049
Day 5	0.175	0.297		0.762	0.184	0.7376	0.1844
Day 7	0.27	0.388	0.762		0.3299	0.799	0.3707
Week 2	0.659	0.416	0.184	0.33		0.0947	0.5328
Week 4	0.136	0.222	0.738	0.799	0.0947		0.1718
Week 9	0.548	0.505	0.184	0.371	0.5328	0.1718	

(B) Jaccard Index

	Arrival	Day 2	Day 5	Day 7	Week 2	Week 4	Week9
Arrival		0.0001	1E-04	4E-04	0.0002	0.0003	0.0001
Day 2	0.0001		0.506	3E-04	0.0003	0.0001	0.0002
Day 5	0.0001	0.506		1E-04	0.0002	0.0002	0.0001
Day 7	0.0004	0.0003	1E-04		0.601	0.0189	0.2071
Week 2	0.0002	0.0003	2E-04	0.601		0.0607	0.1437
Week 4	0.0003	0.0001	2E-04	0.019	0.0607		0.0225
Week 9	0.0001	0.0002	1E-04	0.207	0.1437	0.0225	

Adult B6J CON2

(A) Bray-Curtis Index

	Arrival	Day 2	Day 5	Day 7	Week 2	Week 4	Week9
Arrival		0.161	0.471	0.335	0.1282	0.1502	0.1237
Day 2	0.161		0.099	0.04	0.4712	0.1805	0.5631
Day 5	0.471	0.099		0.593	0.0962	0.1623	0.1147
Day 7	0.335	0.04	0.593		0.0522	0.0868	0.048
Week 2	0.128	0.471	0.096	0.052		0.2401	0.4937
Week 4	0.15	0.181	0.162	0.087	0.2401		0.6543
Week 9	0.124	0.563	0.115	0.048	0.4937	0.6543	

(B) Jaccard Index

	Arrival	Day 2	Day 5	Day 7	Week 2	Week 4	Week9
Arrival		0.0002	2E-04	1E-04	0.0005	0.0001	0.0003
Day 2	0.0002		0.653	0.881	0.5611	0.4755	0.0211
Day 5	0.0002	0.6534		0.837	0.8215	0.8353	0.0222
Day 7	0.0001	0.8814	0.837		0.5465	0.2055	0.0101
Week 2	0.0005	0.5611	0.822	0.547		0.7433	0.0107
Week 4	0.0001	0.4755	0.835	0.206	0.7433		0.0213
Week 9	0.0003	0.0211	0.022	0.01	0.0107	0.0213	

Supplemental Table 4. Pairwise tables comparing time points of other mouse groups during acclimation. (A) Bray-Curtis and (B) Jaccard index pairwise tables comparing arrival through week 9 time points. Boxes in red indicate significant ($p<0.05$) differences between time points. Groups of mice are denoted above tables.
