# Supplementary material for: Acclimation and Institutionalization of the Mouse Microbiota Following Transportation
Source: Front Microbiol. 2018 May 28;9:1085. doi: 10.3389/fmicb.2018.01085 (PMC5985407; doi:10.3389/fmicb.2018.01085)
Supplement: Supplementary file 16 [file Table_5.PDF]

(A) Bray-Curtis Index

(B) Jaccard Index

Adult CJ CON

	Pre-Ship	Facility 1	Facility 2
Pre-Ship		0.002	0.0739
Facility 1	0.002		0.0562
Facility 2	0.0739	0.0562	

	Pre-Ship	Facility 1	Facility 2
Pre-Ship		0.0055	0.0019
Facility 1	0.0055		0.4374
Facility 2	0.0019	0.4374	

Wean B6J CON

	Pre-Ship	Facility 1	Facility 2
Pre-Ship		0.0062	0.0672
Facility 1	0.0062		0.119
Facility 2	0.0672	0.119	

	Pre-Ship	Facility 1	Facility 2
Pre-Ship		0.0016	0.0018
Facility 1	0.0016		0.0279
Facility 2	0.0018	0.0279	

Adult B6J BAR

	Pre-Ship	Facility 1	Facility 2
Pre-Ship		0.1704	0.2974
Facility 1	0.1704		0.3756
Facility 2	0.2974	0.3756	

	Pre-Ship	Facility 1	Facility 2
Pre-Ship		0.0027	0.0033
Facility 1	0.0027		0.5435
Facility 2	0.0033	0.5435	

Adult B6J CON2

	Pre-Ship	Facility 1	Facility 2
Pre-Ship		0.0272	0.0829
Facility 1	0.0272		0.0312
Facility 2	0.0829	0.0312	

	Pre-Ship	Facility 1	Facility 2
Pre-Ship		0.0018	0.0019
Facility 1	0.0018		0.0269
Facility 2	0.0019	0.0269	

Supplemental Table 5. Pairwise tables of institutionalization of other mouse groups. (A) Bray-Curtis and (B) Jaccard index pairwise tables comparing pre-shipping samples to each facility at nine weeks. Boxes in red indicate significant ($p<0.05$) differences between time points. Groups of mice are denoted above tables.
